# Error in Author Name and Affiliations

**DOI:** 10.1001/jamahealthforum.2026.0775

**Published:** 2026-03-27

In the Special Communication titled “Health Systems Approaches for Advancing Implementation and Policy for Food Is Medicine,”^1^ published online on February 20, 2026, the name of the fifth author, Robert J. Ostfeld, MD, did not include a middle initial, and affiliations have been added for the first and eighth authors, Priyansh P. Shah, MD, and Robert T. Faillace, MD. This article was corrected online.
